# Optimization Design of Fluoro‐Cyanogen Copolymer Electrolyte to Achieve 4.7 V High‐Voltage Solid Lithium Metal Battery

**DOI:** 10.1002/advs.202400466

**Published:** 2024-06-18

**Authors:** Weijian Xu, Weiliang Dong, Jianzhou Lin, Kexin Mu, Zhennuo Song, Jiji Tan, Ruixue Wang, Qiang Liu, Caizhen Zhu, Jian Xu, Lei Tian

**Affiliations:** ^1^ Institute of Low‐Dimensional Materials Genome Initiative College of Chemistry and Environmental Engineering Shenzhen University Shenzhen 518060 China; ^2^ Department of Mechanical Engineering The Hong Kong Polytechnic University Hong Kong 100872 China

**Keywords:** 4.7 V high voltage, fluoro‐cyanogen copolymer, LCO, lithium‐metal battery, polymer electrolyte

## Abstract

Raising the charging voltage and employing high‐capacity cathodes like lithium cobalt oxide (LCO) are efficient strategies to expand battery capacity. High voltage, however, will reveal major issues such as the electrolyte's low interface stability and weak electrochemical stability. Designing high‐performance solid electrolytes from the standpoint of substance genetic engineering design is consequently vital. In this instance, stable SEI and CEI interface layers are constructed, and a 4.7 V high‐voltage solid copolymer electrolyte (PAFP) with a fluoro‐cyanogen group is generated by polymer molecular engineering. As a result, PAFP has an exceptionally broad electrochemical window (5.5 V), a high Li^+^ transference number (0.71), and an ultrahigh ionic conductivity (1.2 mS cm^−2^) at 25 °C. Furthermore, the Li||Li symmetric cell possesses excellent interface stability and 2000 stable cycles at 1 mA cm^−2^. The LCO|PAFP|Li batteries have a 73.7% retention capacity after 1200 cycles. Moreover, it still has excellent cycling stability at a high charging voltage of 4.7 V. These characteristics above also allow PAFP to run stably at high loading, showing excellent electrochemical stability. Furthermore, the proposed PAFP provides new insights into high‐voltage resistant solid polymer electrolytes.

## Graphical abstract

A 4.7 V high‐voltage solid copolymer electrolyte (PAFP) containing fluoro‐cyanogen group with ultrahigh ionic conductivity (1.2 mS cm^−2^) at 25 °C, high Li^+^ transference number (0.71) and superior electrochemical window (5.5 V), is designed and synthesized by polymer molecular engineering. These allow superior interface stability (2000 h at 1 mA cm^−2^), and ultra‐stable long‐cycle performance under high voltage and high loading.

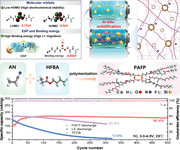

## 1 Introduction

With the rapid development of strategic high‐tech technologies such as the deep sea, deep earth, deep space, and deep blue, the pressing demand for high‐energy‐density batteries is growing with each passing day.^[^ ^1^ ^]^ In particular, with the emergence of electric aircraft such as electric vertical takeoff and landing (eVTOL), flying cars, and other concept products, there is an imperative need for high‐performance batteries to support their long‐range flight.^[^ ^2^ ^]^ However, the commercialized liquid lithium batteries at this phase are not only challenging to achieve the ideal high energy density, but also have severe security risks due to the flammable and explosive liquid electrolyte.^[^ ^3^ ^]^ Therefore, the development of next‐generation solid‐state batteries has become a competitive field for researchers and significant new energy companies around the world.^[^ ^4^ ^]^

In solid‐state battery systems, solid‐state electrolyte is a crucial technology because it changes the interface contact with the electrode.^[^ ^5^ ^]^ At present, inorganic solid electrolyte (ISE)^[^ ^6^ ^]^ and solid polymer electrolyte (SPE)^[^ ^7^ ^]^ have been extensively studied. Among them, ISE possesses a high room temperature conductivity (>10^−4^ S cm^−2^) and a broad electrochemical window, but its poor contact with the electrode interface, high sensitivity to oxygen and water, coupled with inherent brittleness, poor interface expansibility, and other characteristics restrict its further development.^[^ ^8^ ^]^ SPE's high flexibility and machinability, superior mechanical properties, low density, and high electrode/solid electrolyte interface contact area make them even more advantageous.^[^ ^9^ ^]^ However, due to the low ionic conductivity of SPEs at room temperature (<10^−4^ S cm^−2^),^[^ ^10^ ^]^ the narrow electrochemical window, and the poor electrochemical stability, the preparation of high energy density solid polymer batteries with SPE matching high‐pressure positive electrode is still a great challenge.^[^ ^9^, ^11^ ^]^

LiCoO_2_ (LCO) has become a leader in cathode materials for lithium‐ion batteries due to its advantages of ultra‐high vibration density, steady voltage, and simple mass production. However, when it operates at a voltage >4.55 V, its performance deteriorates rapidly due to structural collapse induced by metastable phase and surface lattice oxygen release.^[^ ^12^ ^]^ In addition, it has been pointed out that the polymer electrolyte is hard to match the high‐voltage positive electrode, so the construction of LCO solid polymer batteries is still a major problem.^[^ ^13^ ^]^ To solve the above problems, the primary strategy is to increase the electrochemical window of the polymer electrolyte and form a stable interface layer. The specific approach predominantly originates with the molecular structure and introduces different functional groups. Based on this, ciucci^[^ ^14^ ^]^ introduced fluoride functional groups (─F) to promote the formation of fluorine‐rich interface phase, enhance the interface stability, and effectively inhibit the growth of lithium dendrites. Chen^[^ ^15^ ^]^ introduced a fluorine crosslinker to decrease the electron cloud density of oxygen‐containing polar groups and enhance the oxidative stability of the polymer electrolyte. The cyano group (─CN) possesses a significant electronegativity, a low HOMO value, and forms chelating coordination with lithium ions, contributing to the dissociation of lithium salts. In addition, the reduction of the cyanide group will form a stable and uniform SEI^[^ ^16^ ^]^ on the lithium metal side. Cui et al. designed and synthesized a class of lithium borate cyanide and a new cyano siloxane multifunctional additive to enhance the high‐voltage cycle life of the entire battery, which demonstrates that ─CN can form a thin and robust CEI film to stabilize the cathode material and inhibit the dissolution of effective transition metals.^[^ ^17^ ^]^ However, few polymer electrolytes capable of matching LCO positivity above 4.6 V have been recorded so far.

Herein, a copolymer electrolyte network containing fluoro‐cyanogen group (PAFP) was designed and synthesized by utilizing the molecular engineering strategy of polymer materials. The cross‐linked network of fluoro‐cyanogen is beneficial to the electrochemical stability and transport of the electrolyte, resulting in an ultrahigh ionic conductivity (1.2 mS cm^−2^) at room temperature, a high Li^+^ transfer number (*t*_Li+_) of 0.71, and a wide electrochemical window of 5.5 V. These characteristics enable the Li||Li symmetric battery to experience stable Li stripping/plating behavior over 2000 h at 1 mA cm^−2^, while matching the high voltage positive LCO. Moreover, the maximum capacity and capacity retention of assembled LCO|PAFP|Li cells are 178.8 mAh g^−1^ and 73.7% at 1 C, and 4.5 V, respectively. Their normal operation even under high loading and high voltage (4.7 V) demonstrates the superior electrochemical stability of PAFP copolymer electrolyte. More significantly, our proposed PAFP provides a new scheme for improving electrochemical stability and Li^+^ transport of solid electrolytes.

## 2 Results and Discussion

**Figure 1:**
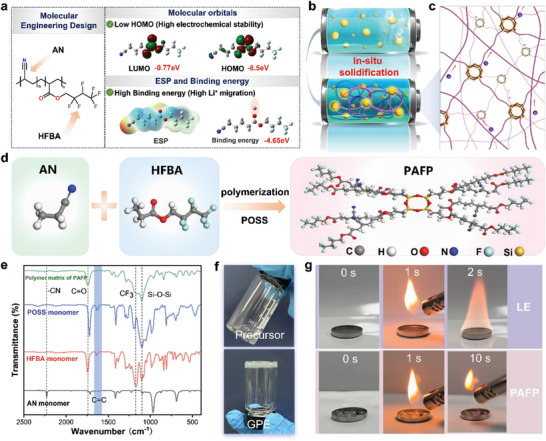
Molecular engineering design, preparation, and characterization of PAFP. a) Schematic illustration of the designed high voltage and high Li^+^ migration. b) Schematic diagram of the design principle of in situ cross‐linked polymer electrolyte. c) Schematic representation of the structure of the PAFP cross‐linking network. d) Schematic of the polymer synthesis design. e) FTIR spectra of synthetic PAFP. f) Image of the precursor solution cured in situ into a gel polymer electrolyte. g) Combustion tests of PAFP and LE electrolytes.

The challenges of ionic conductivity, interface, and high voltage cycle instability of polymer electrolytes considerably hinder their development in the field of solid‐state batteries.^[^ ^18^ ^]^ In addition, under high voltage, the electrolyte is prone to oxidative decomposition, and the cycle of the battery is reduced.^[^ ^19^ ^]^ Therefore, how to make polymer electrolytes withstand high voltage and have high ionic conductivity is a critical challenge to accomplishing higher energy density solid‐state batteries.^[^ ^20^ ^]^ To address the dilemma of low Li^+^ transport and electrochemical stability, it is absolutely essential to synthesize high‐performance polymer electrolytes from the perspective of polymer molecular engineering design. The molecular engineering strategy can simulate its electrochemical stability and Li^+^ transport by calculating its molecular orbital and binding energy by density functional theory (DFT) as shown in **Figure** **1**a. Low HOMO (Highest Occupied Molecular Orbital) molecules can further stabilize the high‐voltage cathode.^[^ ^21^ ^]^ The high binding capacity of Li^+^ can indicate its potent capability to dissociate Li^+^. The results of the calculation demonstrate that the monomers acrylonitrile (AN) and 2,2,3,4,4,4‐hexafluorobutyl acrylate (HFBA) have lower HOMO (below −8 eV), which can further stabilize the cathode and can be matched with the high voltage cathode (Figure S1, Supporting Information). However, the HOMO and LUMO of AN are relatively low, indicating that its compatibility with Li metal is poor, while HFBA possesses good compatibility with lithium metal. Therefore, it is necessary to solve the problem of high voltage and compatibility of molecules by further copolymerization. The copolymerized fluorine has better LUMO, HOMO, and binding energy with Li^+^. From the theoretical calculation, the high voltage bearing force and high ionic conductivity electrolyte demand to have ─CN, ─CF_3_, and OCOR groups. Azodiisobutyronitrile (AIBN) was applied as initiator, (fluoride carbonate) FEC as a solvent, and AN, HFBA, and acrylic POSS cage mixture (POSS) as a monomer for in situ radical polymerization (Figure 1b). The lithium salts are lithium difluoromethane sulfonimide and difluoroxalate borate (molar ratio: 5:1). The electrolyte with a strong cross‐link network structure was synthesized as PAFP (Figure 1c). When POSS is used as a cross‐linking agent, the terminal chains of POSS with eight acrylate groups can be connected to the copolymer molecular chains of AN and HFBA by free radical polymerization (Figure 1d), forming an enormous 3D cross‐linking network. Fourier transform infrared (FTIR) spectroscopy was used to confirm the radical polymerization of AN, HFBA, and POSS in Figure 1e. In the PAFP matrix, the characteristic peak of the C═C double bond at 1630 cm^−1^ disappeared and the characteristic peaks of C─F and Si─O─Si appeared, indicating successful polymerization. Thus, the cross‐linked network in three dimensions supplies rigidity and steadiness to the electrolyte.

In addition, the addition of FEC, on the one hand, is conducive to the migration of Li^+^. On the other hand, it can further stabilize the Li metal interface, which can make SPE have excellent firmness. Figure 1f shows a digital photograph of the polymerization of the PAFP precursor solution into a gel electrolyte. It can be plainly seen that the precursor solution becomes gel after passing through the polymer. The electrolyte scanning electron microscope (SEM) image of PAFP revealed that the glass cellulose separator was completely penetrated into the membrane space by PAFP. PAFP thickness ranges from 260 to 280 µm (Figure S2, Supporting Information). More grandly, the safety performance of polymer electrolytes is also a problem that cannot be neglected. Thermal gravimetric analysis (TGA) was used to study the thermal stability of polymer electrolytes (Figure S3, Supporting Information). The TGA curve of PAFP exhibits a favorable plateau, at the same time sample begins to decompose when the temperature reaches 245 °C, which can be attributed to the beginning degradation of the polymer chain, indicating the high thermal stability of the PAFP. To analyze the flame retardant performance of PAFP, the self‐extinguishing time of PAFP and LE electrolyte during combustion was tested. The results are shown in Figure 1g, Videos S1 and S2 (Supporting Information). The LE electrolyte burns immediately upon contact with the flame. In sharp contrast, the PAFP did not ignite. Therefore, PAFP has a good flame‐retardant effect. Free radicals during HO· combustion are measured by electron spin resonance (ESR) (Figure S4, Supporting Information). The aggregate number of HO· radicals that can burn in the gas phase LE is lower, which proves that PAFP cannot burn in the gas phase.^[^ ^22^ ^]^

### 2.1 Li^+^ Transport Performance

**Figure 2:**
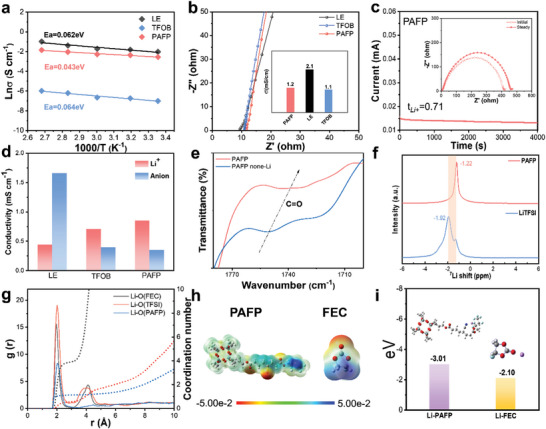
Li^+^ transport performance. a) Arrhenius plots of ionic conductivity of PAFP. b) Nyquist curves of PAFP, TFOB, and LE and corresponding ionic conductivity at room temperature. c) Polarization curves, initial and steady‐state impedance maps of PAFP at room temperature. d) Histogram of Li^+^ and anion conductivity for PAFP, TFOB, and LE. e) FTIR spectra of PAFP and PAFP without LiTFSI. f) 7Li NMR spectrum of LiTFSI in PAFP. g) Radial Distribution Function (RDF) and coordination number (CN) of Li and O in PAFP, TFOB, and LE. h) Electrostatic potential profiles of PAFP and FEC. i) Binding energies of Li^+^ with PAFP and FEC.

Possessing high ion transport performance is a critical index to acquiring a high‐performance solid‐state battery. Differential scanning calorimetry (DSC) and X‐ray diffraction (XRD) were used to study the glass transition temperature and crystalline phase of the polymer (Figures S5 and S6, Supporting Information). PAFP has a low glass transition temperature of −28 °C and no crystalline phase, which is conducive to the migration of Li^+^ in PAFP.^[^ ^23^ ^]^ So as to further study the ionic conductivity of the electrolyte, the electrochemical impedance spectroscopy (EIS) was tested for PAFP, TFOB, and LE electrolyte at different temperatures (Figure S7, Supporting Information). As can be seen from **Figure** **2**a, their ionic conductivity increases approximately linearly with the increase in temperature. The related activation energy can be calculated by the Aranius equation. PAFP has a low activation energy (0.043 eV), which signifies that PAFP possesses a low energy barrier for Li^+^ migration and can migrate rapidly in the electrolyte.^[^ ^24^ ^]^ Figure 2b displays the impedance curves of PAFP, TFOB, and LE at room temperature. The ionic conductivity of the corresponding electrolyte at room temperature is shown in the inset. The cross‐linking network in PAFP has a large binding energy for anions. Furthermore, the migration of Li^+^ and the dissociation of lithium salts are facilitated by the abundant carbonyl group (O═C). Li^+^ is moved in this structure via both the interaction between the polymer's polar groups and the rapid transport channel of FEC. As a result, PAFP has a high ionic conductivity of 1.2 mS cm^−1^, which is close to LE (2.1 mS cm^−1^). The conductivity of ions is also significantly influenced by the mobility number of Li^+^. The migration number can be calculated via the Bruce‐Vincent Evans equation. Figure 2c shows that PAFP has a Li+ migration number of 0.71, which is higher than that of LE and TFOB electrolytes (Figure S8, Supporting Information). Furthermore, Figure 2d shows the migration of Li^+^ and anions in the ionic conductivity of the electrolyte. PAFP restricts the movement of anions due to its huge cross‐link network structure. The findings demonstrate that the conductivity of Li+ in PAFP is potent, demonstrating the fast Li transmission performance of PAFP.

In addition, the abundant polar groups (C═O) in PAFP facilitate the migration of Li+ dissociated from lithium salts, which is also the high transport performance of PAFP. Dipolar interactions between lithium ions and C═O groups can be observed using FTIR infrared spectroscopy. As shown in Figure 2e, compared with PAFP‐none Li, the wave number of the C═O group characteristic peak of PAFP is reduced and the wavelength is redshifted, which proves the interaction between PAFP and Li^+^.^[^ ^25^ ^]^ Besides, anions in lithium salts are easy to anchor into the cross‐linked polymer network, which can promote the increase of Li^+^ migration number. At the same time, these interactions also contribute to electrochemical stability. The conduction mechanism of Li in the PAFP electrolyte was explored by solid‐state ^7^Li nuclear magnetic resonance (NMR) spectroscopy (Figure 2f). Compared with the pure LiTFSI, the ^7^Li NMR peak of the PAFP electrolyte is shifted by 0.7 ppm to the lower field. This shift to the lower field is attributed to the anchoring interaction between PAFP and Li^+^, which reduces the electron density around the Li.^[^ ^26^ ^]^

Molecular dynamics (MD) simulations were used to verify the Li+ transport mechanism in SPE. Snapshots of MD simulations (Figure S9, Supporting Information), radial distribution function (RDF), and coordination number (CN) of PAFP are shown in Figure 2g. The RDF between Li^+^ and O of PAFP shows a sharp peak at 2.07 Å with a CN of 1.13, while the coordination peak between Li^+^ and O of TFSI appears at 2.02 Å with a CN of 3.03. Additionally, Li^+^ and O in FEC are 1.97 Å, and CN is 3.91. These simulation results show that Li^+^ coordinates with C═O in PAFP and FEC. Li^+^ co‐transports by interacting with the C═O group of PAFP and FEC molecules.^[^ ^27^ ^]^ DFT calculations were performed to investigate further the strength of the interaction between solvent molecules and Li^+^ and polymer. The electron density distribution around PAFP and FEC is seen from the electrostatic potential (ESP) (Figure 2h). The oxygen atoms in PAFP and FEC molecules have a high charge density and are readily coordinated with Li^+^.^[^ ^28^, ^29^ ^]^ Compared to Li‐FEC (−2.10 eV), Li‐PAFP (−3.01 eV) has a higher binding energy, implying that Li^+^ exhibit significant interaction with the PAFP network. This coordination can direct the migration of Li and effectively restrain the deposition of Li^+^ on the negative electrode surface.^[^ ^30a^ ^]^ At the same time, this also reveals that PAFP has a high Li^+^ conduction ability, which can induce rapid migration and uniform deposition of Li^+^.

### 2.2 Interface Stability and Evolutionary Mechanism of PAFP

**Figure 3:**
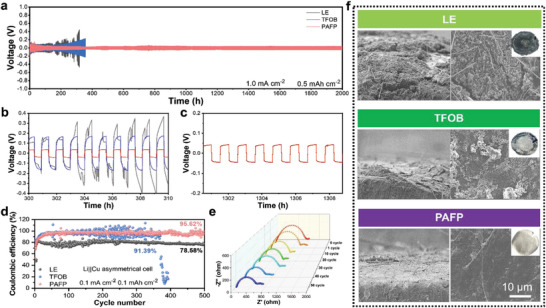
Compatibility with lithium metal interface. a) Galvanostatic cycling curves of Li||Li symmetric cells with PAFP, TFOB, and LE at 1 mA cm^−2^ and 0.5 mAh cm^−2^. b) Enlarged view of the 300–310 h overpotential of PAFP, TFOB and LE. c) Enlarged view of the 1300–1310 h overpotential of PAFP. d) The corresponding Coulombe efficiencies of PAFP, TFOB, and LE at a current density of 0.1 mA cm^−2^ and a lithium plating capacity of 0.1 mAh cm^−2^. e) In situ EIS of PAFP Li||Li symmetric cell at a current density of 1 mA cm^−2^. f) SEM surface morphology and cross‐sectional view of PAFP, TFOB, and LE assembled Li||Li symmetric cells after 50 cycles at 1 mA cm^−2^, 0.5 mAh cm^−2^ (the insets show digital photos of the surface morphology of the corresponding lithium metal used by the electrolyte after the cycle).

Furthermore, to probe the compatibility between the electrolyte and Li metal, the Li||Li symmetric battery was assembled and tested under the condition of a current density of 1 mA cm^−2^ and a fixed capacity of 0.5 mAh cm^−2^ (**Figure** **3**a). At a high current density of 1 mA cm^−2^, the polarization voltage of LE and TFOB gradually increased, generating a short circuit phenomenon. At the same time, the polarization voltage of PAFP reaches ≈50 mV (typical amplified polarization voltage curves Figure 3b,c) for >2000 h without significant fluctuation and short circuit, which indicates PAFP has exceptional compatibility with Li metal. Otherwise, the stable performance at 0.5 mA cm^−2^ is >1200 h, indicating that it is still compatible with lithium metal at low current density (Figure S10, Supporting Information). In addition, constant current charging and discharging tests of PAFP, TFOB, and LE were carried out at current densities ranging from 0.1 to 2 mA cm^−2^ (Figures S11–S13, Supporting Information). With the current increase, the voltage increases accordingly. TFOB and LE have been short‐circuited at 0.5 mA cm^−2^. Unexpectedly, PAFP is still steady at a high current density of 2 mA cm^−2^, obtaining a satisfactory magnification performance.

Moreover, the average lithium plating/stripping Coulomb efficiency ability of electrolytes PAFP, TFOB, and LE was tested by assembling Li||Cu cells, as shown in Figure 3d. The Li||Cu assembled by PAFP demonstrated adequate Coulomb efficiency (95.62%). Compared with LE (78.58%) and TFOB (91.39%), PAFP exhibited higher reversibility of stripping and Li plating. It is encouraging that the in‐situ EIS of Li||Li symmetric cells were tested at a current density of 1 mA cm^−2^ to research the interface impedance of PAFP at different cycle numbers (Figure 3e). As the cycle number increases, the interface resistance decreases continuously. After 50 cycles, the interface resistance declines to 420 Ω. This can be ascribed to the formation of a more stable interface layer (Figure 3a).

Scanning electron microscopy (SEM) was employed to study the surface morphology and cross‐section of the Li metal anode after 50 cycles (Figure 3f), and the upper right inset shows the Li metal surface after the contact cycle between the electrolyte and the Li metal. From digital photos, it can be seen that the Li metal surface in contact with LE and TFOB presents dark dead lithium. The SEM snapshot shows that the electrode surface in contact with LE and TFOB possesses a porous and rough surface, while the Li metal surface in contact with PAFP is tighter and flatter, and maintains its due metallic luster. The tighter surface can effectively inhibit the growth of lithium dendrites. It can reduce the occurrence of detrimental side reactions, so that Li^+^ can be transferred evenly, resulting in high electrochemical stability and increased cycle life.^[^ ^31^ ^]^

**Figure 4:**
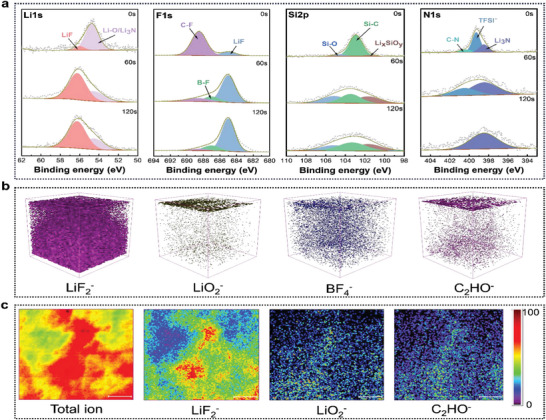
Evolutionary mechanisms for interface stability. a) XPS profiles of PAFP‐based Li||Li symmetric cells at different depths after 25 cycles at a current density of 1 mA cm^−2^. b,c) 3D rendering and high‐resolution lateral chemical mapping of TOF‐SIMS.

X‐ray photoelectron spectroscopy (XPS) inspection of the interface layers at various depths on the Li metal surface after 25 cycles was carried out so as to investigate the mechanism of the stable evolution of the interface in more detail (**Figure** **4**a). Following cycling, Li 1s and F 1s spectra can be clearly detected, with LiF peaks appearing at 56 eV and 685 eV, respectively, which are mainly derived from the decomposition of PAFP, LiTFSI, LiDFOB, and FEC on the Li surface. LiF induced by the decomposition of these molecules on the Li surface is able to inhibit the growth of lithium dendrites, preserve high conductivity, and further stabilize the Li metal interface.^[^ ^32^ ^]^ A new peak of B‐F (687.08 eV) appeared in the F 1s spectrum of 60 s etched, which was attributed to the reduction decomposition of DFOB‐ anion to further stabilize the interface layer. However, as the Si 2p and N 1s spectra demonstrate, the Si─O and ─CN on the PAFP chain can be broken down into LixSiOy^[^ ^33^ ^]^ and Li3N‐rich SEI. These features demonstrate that the polymer chain's silicon and nitrogen groups play a role in the SEI layer's development. Additionally, as the depth grows, rich inorganic systems including LiF, Li_3_N, and LixSiOy evolve exceedingly stronger, confirming that inorganic components like F, N, and Si are abundant within the interior structure of SEI. This accelerates the transport of Li^+^ and induces homogeneous deposition of Li. The strong inorganic layer SEI not only effectively inhibits the growth of lithium dendrites, but also achieves a stable ultra‐long cycle. The primary chemical components of SEI were visually identified using time‐of‐flight secondary ion mass spectrometry (TOF‐SIMS) (Figure 4b,c). LiF_2_^−^, LiO_2_^−^, BF_4_^−^ and C_2_HO were selected as fragments representing the SEI components in 3D rendering (Figure 4b), revealing the presence of these inorganic compounds. However, Figure 4c is a high‐resolution TOF‐SIMS spectrograph of chemical mapping, from which it is clear that the SEI of inorganic components generated based on PAFP is uniform and dense. The firmness and compactness of the SEI layer generated by PAFP can facilitate Li^+^ conduction and stabilize the Li metal surface, as demonstrated by the experimental results.

### 2.3 Superior High Voltage Compatibility for Solid‐State Lithium‐Metal Batteries

**Figure 5:**
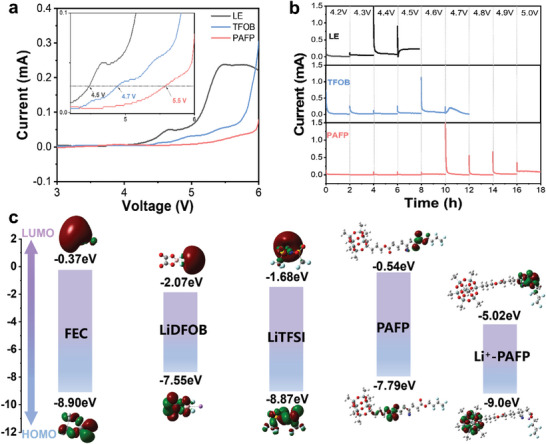
Superior high voltage stability analysis of PAFP. a) Linear sweep voltammetry (LSV) curves of electrolytes for PAFP, TFOB, and LE at a scan rate of 1 mV s^−1^. b) The LCO||Li cell was assembled for electrochemical float testing of PAFP, TFOB, and LE electrolytes. c) DFT calculation plots of molecular orbitals LUMO and HOMO.

A high voltage oxidation window is essential for higher energy density solid‐state lithium metal batteries. Utilizing linear sweep voltammetry (LSV), the intrinsic oxidative stability of PAFP, TFOB, and LE electrolytes was initially examined. The LSV curve (versus Li/Li^+^) from 3 to 6 V is displayed in **Figure** **5**a. The LSV curve can clearly demonstrate that the oxidation decomposition of LE electrolyte occurs at 4.5 V, and the oxidation decomposition of TFOB is at 4.7 V, which proves that the oxidation stability of LE and TFOB is poor. Compared with liquid electrolytes, the oxidation potential of PAFP can reach 5.5 V, and the oxidation stability is significantly improved. This manifests the feasibility of matching PAFP with a high‐voltage cathode. Indicating that designing polymer electrolytes at the molecular level can effectively improve their electrochemical window.

To further characterize the actual oxidative stability of PAFP in the cell, timed current method (CA) experiments were performed with LCO electrode‐assembled LCO||Li cells (Figure 5b). The measured leakage current could directly assess the actual oxidative stability of the electrolyte.^[^ ^34^ ^]^ The leakage current directly increases when the voltage of the LE liquid electrolyte climbs to 4.5 V, confirming that the LE electrolyte is readily oxidized. In addition, the leakage current of liquid TFOB electrolytes also increases when the voltage is added to 4.7 V, indicating that TFOB is unstable at high voltage. In stark contrast, even when the voltage is raised to 5.0 V, the leakage current of the PAFP electrolyte does not increase noticeably. It is still in a very low state, which fully demonstrates its superior stability to a high‐voltage cathode. Posteriorly, the oxidative stability of PAFP was verified by theoretical calculation of DFT. The HOMO of PAFP is comparatively low (−7.79 eV), displayed in Figure 5c. The HOMO of PAFP exhibits an unexpected −9.0 eV when it interacts with Li^+^, further demonstrating the material's exceptional high‐pressure stability. The electrochemical window can be efficiently enhanced by the molecular design of polymers, offering inspiration for the development of high‐energy density solid‐state battery designs.

**Figure 6:**
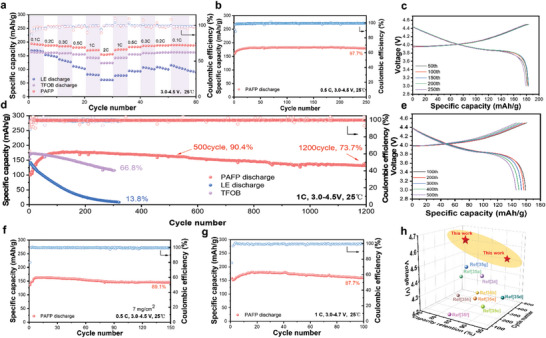
Electrochemical performance of SPE. a) Rate performance of electrolytes PAFP, TFOB, and PAFP in LCO||Li cells. b) Long cycle performance of LCO|PAFP|Li cells at 0.5 C, 4.5 V. c) Charge and discharge curves of LCO|PAFP|Li cells at 0.5 C. d) Cycle performance of LCO||Li cells assembled with PAFP, TFOB and LE electrolytes at 1 C, 4.5 V. e) Charge and discharge curves of LCO|PAFP|Li cells at 1 C. f) Cycling performance of LCO|PAFP|Li cells at high loading cathode, 4.5 V, 0.5 C. g) Cycling performance of LCO|PAFP|Li cells at 1 C, 4.7 V high voltage. h) With recent reports of polymer LCO||Li battery voltage, cycle number, and the comparison of retention capacity.

Due to the high electrochemical window of PAFP, it is able to match with the high‐voltage cathode. Therefore, a higher specific cell capacity can be obtained by coordinating a high‐voltage cathode such as LCO. PAFP to 0.1 C and 0.2 C and 0.3 C and 0.5 C and 1 C, 2 C shows under the ratio of 189.2, 187.7, 184.9, 180.2, 170.4, and 155.6 mAh g^−1^ capacity, high rate shows that LCO|PAFP|Li has superior reversible (**Figure** **6**a). It is worth noting that when the current density is returned from 0.1 C to 0.1 C, the capacity is comparable to the initial capacity, demonstrating the good rate performance of LCO|PAFP|Li (Figure S14, Supporting Information). At a low current density of 0.5 C, the capacity of the PAFP assembled battery can reach 182.4 mAh g^−1^ (Figure 6b,c). After 250 cycles, its capacity retention rate is 97.7%, indicating that the battery assembled by PAFP has ultra‐high stability compared to LE and TFOB at 4.5 V high voltage (Figures S15 and S16, Supporting Information). The stability of long‐cycle performance at a high rate is also the basis for evaluating the superior performance of the battery.

Figure 6d shows the cycle performance of the LCO|PAFP|Li cell at room temperature in the voltage range of 1 C, 3.0–4.5 V. The capacity retention rate of the LE‐based battery is only 13.8% after 320 cycles of high voltage cycle. Unfortunately, the TFOB battery also decays from the initial 155 to 115 mAh g^−1^ after 303 cycles, with a capacity retention of 66.8%. Rapid electrode and electrolyte consumption results from the unchecked parasitic reactions of liquid electrolytes LE and TFOB. Especially in the case of high voltage, the electrolyte with poor electrochemical stability accelerates the battery capacity decay.

This uncovers that it is difficult for LE and TFOB to match the high‐voltage cathode. In comparison, the maximum capacity of the PAFP battery is 178.8 mAh g^−1^. Surprisingly, at a cutoff voltage of 4.5 V, the capacity retention rate of the battery reaches 73.7% for 1200 cycles, demonstrating the reliable stability of the PAFP. (Figure 6e). The reduction of interface impedance enhances the transportation of lithium ions in the interface phase, leading to a gradual increase in battery capacity after multiple cycles (Figure S17, Supporting Information). Also encouraging, we investigated the electrochemical performance of the 4.5 V LCO|PAFP|Li full cell when using a highly loaded cathode (Figure 6f). The PAFP cell assembled using a high‐load LCO cathode of 7 mg cm^−2^ showed high stability after 150 cycles with a retention rate of 89.1% (Figure 6g). It is noteworthy that after 100 cycles at 1 C 4.7 V high voltage, the capacity retention rate is 87.7%. Benefiting from the high oxidative properties of PAFP, PAFP is able to circulate stably under high voltage and high loading (Figure S18, Supporting Information). The entire demonstration of PAFP under high pressure and high loading LCO||Li batteries in real application potential is provided by the experimental results. The impressive high voltage stability exhibited by LCO|PAFP|Li cells demonstrates superior high voltage cycling performance compared to previously reported polymer Li metal cells. This is evident from the comprehensive comparison of cutoff voltage, number of cycles, and capacity retention ratio as shown in Figure 6h and Table S1 (Supporting Information).^[^ ^30^, ^34^ ^]^

### 2.4 Electrochemical Stability Mechanism of PAFP

**Figure 7:**
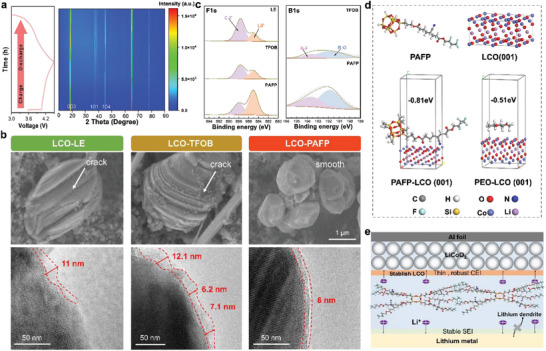
Electrochemical characterization of the LCO cathode material after cycling. a) In situ XRD of LCO cathode during charge‐discharge cycle in PAFP electrolyte. b) SEM and TEM images of LCO after cycling in PAFP, TFOB, and LE electrolytes. c) XPS analysis image of the LCO interface. d) Binding energy between PAFP, PEO, and LCO cathode. e) Schematic diagram of the principle of PAFP to maintain high performance under high voltage.

The LCO|PAFP|Li cell was assembled for in‐situ XRD characterization to investigate its structural evolution during a 4.5 V cutoff voltage (**Figure** **7**a). There is no discernible offset during the charging and discharging process, based on the distinctive diffraction peaks (003), (101), and (104) of LCO‐PAFP. This proves that PAFP can effectively suppress the irreversible intercrystalline cracks and phase transitions of LCO. The high‐voltage cathode interface compatibility with PAFP is responsible for the SSBs' excellent capacity retention rate.^[^ ^35^ ^]^ To confirm the LCO's altered crystal structure, transmission electron microscopy (TEM) and SEM were employed (Figure 7b). Compared with the LCO before cycling (Figure S19, Supporting Information), the LCO particles containing LE and TFOB were clearly cracked. The fractured LCO hastened capacity attenuation, demonstrating its instability under the high‐voltage electrode and jeopardizing the battery's long‐term stability.^[^ ^36^ ^]^ However, the PAFP‐based LCO particles show a relatively smooth and flat surface, which also verifies their high voltage stability.

Furthermore, following TEM cycling, we monitored the interface evolution of the LCO cathode. It is distinct to detect the PAFP electrolyte forms a uniform dense CEI layer. In contrast, unstable CEI layers can be observed in conventional liquid electrolytes LE and TFOB. XPS tests were also performed to analyze the chemical composition of the LCO cathode surface (Figure 7c). The examination of the F 1s, Li 1s, and B 1s spectra reveals that the majority of the inorganic materials that make up the CEI layer of the PAFP‐based LCO electrode are rich in B─F, B─O, and LiF. It fully shows that the cathode interface layer of PAFP can form inorganic substances. It has been demonstrated that it can both form a stable high‐voltage LCO interface to boost Li^+^ conductivity and prevent the electrolyte from breaking down during high‐voltage cycles.^[^ ^37^ ^]^ The interaction between PAFP, PEO, and LCO (001) was investigated by DFT calculations (Figure 7d). Depending on the outcomes, it may be further hardened the high voltage cathode that PAFP and LCO (001) have a greater binding energy (−0.81 eV) than the conventional PEO SPE (−0.51 eV).^[^ ^38^ ^]^ PAFP designed by polymer molecular engineering can harden the cathode and form a stable SEI with Li metal, effectively inhibiting the growth of Li dendrites, which is also the reason for maintaining a high retention rate during the high‐voltage long cycle (Figure 7e).

## 3 Conclusion

In conclusion, we have designed a fluoro‐cyanogen copolymer molecule (PAFP) with low HOMO, high binding energy, and resistance to 4.7 V high voltage by polymer molecular engineering. Fast Li^+^ conduction allows the PAFP to possess a large electrochemical window of 5.5 V, a high Li^+^ transference number (0.71), and superior ionic conductivity (1.2 mS cm^–2^). Abundant ─CN and C─F can induce homogeneous deposition of Li^+^, forming a solid SEI layer with LiF, Li^3^N, and LixSiOy as the main inorganic components. Meanwhile, the antioxidant CEI with rich fluorine and boron is constructed to reduce the structural change of LCO and the consumption of electrolytes at high voltage and maintain the satisfactory stability of the battery. Therefore, the Li||Li symmetric battery exhibits excellent reversibility of stripping/plating Li, with a stable cycle of 2000 h at a current density of 1 mA cm^−2^. The assembled LCO|PAFP|Li battery exhibits superior stability at 4.5 V high voltage, with a capacity retention rate of 73.7% at 1200 cycles at 1 C. In addition, even at 4.7 V high voltage, there is still 87.7% capacity retention after 100 cycles at 1 C, demonstrating fantastic high voltage stability. Thus, this work provides an important solution for polymer‐based high‐voltage solid‐state electrolyte materials.

## Conflict of Interest

The authors declare no conflict of interest.

## Supporting information

Supporting Information

Supplemental Video 1

Supplemental Video 2

## Data Availability

The data that support the findings of this study are available from the corresponding author upon reasonable request.
