# Supplementary material for: Optimization Design of Fluoro‐Cyanogen Copolymer Electrolyte to Achieve 4.7 V High‐Voltage Solid Lithium Metal Battery
Source: Adv Sci (Weinh). 2024 Jun 18;11(31):2400466. doi: 10.1002/advs.202400466 (PMC11336954; doi:10.1002/advs.202400466)
Supplement: Supplementary file 1 — Supporting Information [file ADVS-11-2400466-s001.pdf]

ADVANCED SCIENCE
Open Access

Supporting Information

for *Adv. Sci.*, DOI 10.1002/advs.202400466

Optimization Design of Fluoro-Cyanogen Copolymer Electrolyte to Achieve 4.7 V High-Voltage Solid Lithium Metal Battery

*Weijian Xu, Weiliang Dong, Jianzhou Lin, Kexin Mu, Zhennuo Song, Jiji Tan, Ruixue Wang, Qiang Liu, Caizhen Zhu, Jian Xu and Lei Tian**

Supporting Information

Optimization Design of Fluoro-Cyanogen Copolymer Electrolyte to Achieve 4.7 V High-Voltage Solid Lithium Metal Battery

Weijian Xu,[1] Weiliang Dong,[1] Kexin Mu,[1] Zhennuo Song,[1] Jiji Tan,[1] Ruixue Wang,[1] Qiang Liu,[2] Caizhen Zhu,[1] Jian Xu,[1] Lei Tian[1]*

[1] Institute of Low-Dimensional Materials Genome Initiative, College of Chemistry and Environmental Engineering, Shenzhen University, Shenzhen, 518060, China

[2] Department of Mechanical Engineering, The Hong Kong Polytechnic University, Hong Kong, 100872, China

***Corresponding author:**

E-mail: leitian@szu.edu.cn

Shenzhen University, 3688 Nanhai Avenue, Shenzhen 518060, China.

1. Experimental section

Chemical reagents

2,2,3,4,4,4-Hexafluorobutylacrylate (HFBA, 99%), Acrylonitrile (AN, 99%), Azodiisobutyronitrile (AIBN, 98%) were purchased from Shanghai Macklin Biochemical Co., Ltd. Lithium difluoro (oxalato) borate (LiDFOB), bistrifluoromethanesulfonimide lithium salt (LiTFSI), Liquid electrolyte (LE) consists of 1.0 M $LiPF_6$ in EC (Canrd, 99.99%): DMC (Canrd, ≥99.9%): DEC (Canrd, 99.99%) =1:1:1 vol% are purchased from Guangdong Canrd New Energy Technology Co., Ltd. All reagents were used as received without further purification.

Preparation and synthesis of electrolytes

Electrolyte preparation and synthesis: FEC electrolyte with a concentration of 1.2 mol/L LiTFSI: LiDFOB (molar ratio 5:1), denoted as TFOB, was prepared. The precursor solution was formed by dissolving monomer AN/HFBA (1:5, V/V), 5%POSS and initiator 1%AIBN in 70vol% TFOB. The precursor solution was added drop by drop to a glass cellulose separator with a thickness of 260 μm (GF/C, whatman) for in situ polymerization at 70°C for 4 h, denoted as PAFP.

Cathode and battery preparation.

$LiCoO_2$ (LCO) cathodes were prepared by $LiCoO_2$, Super P and polyvinylidene fluoride were mixed and stirred in NMP solvent at a weight ratio of 8:1:1 for 12 h to form a uniform and stable slurry, and the slurry was evenly coated on the aluminum

foil. The cathode was dried in a vacuum oven at 70°C for 12 h to remove the NMP solvent. The load of the prepared $LiCoO_2$ cathode is about 1.0 mg cm^{-2}-1.5 mg cm^{-2}. All coin cells used were CR2032 type, and their assemblies were conducted in an argon-filled glove box (H_2O < 0.1 ppm, O_2 < 0.1 ppm). The charge/discharge tests of coin-type cells (CR2032) were conducted on LAND testing system (Wuhan LAND electronics Co., Ltd.).

Materials characterization

The morphologies of the sample were examined by a scanning electron microscope (S-3400N, HITACHI). FTIR spectra using Bruker Germany to investigate the structure of the electrolytes. The thermal stability of solid electrolyte was monitored by differential scanning calorimetry (METTLER, Switzerland) and thermogravimetric analysis (METTLER, Switzerland) under a nitrogen flow with a heating rate of 10 °C/min. XPS was tested by the Thermo Scientific K-Alpha+ (Thermo Fisher Scientific). The TEM was tested by the Japanese electronics company JEM-2100 & X-Max80. Hydroxyl radicals were tested by an electron spin resonance spectrometer ESR, model Bruker A300.

Electrochemical characterization

Electrochemical impedance spectroscopy (EIS) tests were conducted at the Shanghai Chenhua Instrument Co., LTD in a frequency range from 0.1 Hz to 100 kHz. The testing temperatures were ranged from 25 to 100℃ and the ionic conductivity σ is

calculated by the following equation:

$$\sigma = \frac{L}{SR} \qquad (1)$$

where L represents the thickness (cm) of the electrolyte membrane, R represents the bulk resistance (Ω), and S corresponds to the contact area (cm^2) between SS and electrolyte. The impedance spectra were measured by scanning in a frequency range from 0.1 Hz to 100 kHz in the symmetrical Li|Li cells.

LSV measurements were conducted at a sweep rate of 1 mV s^{-1} from 2 to 6 V.

The Li-ion transfer number (t_{Li}^{+}) was calculated by the following equation:

$$t_{Li+}=(I_{ss}\times R_{bss}\times(\Delta V-I_0\times R_{i0}))/(I_0\times R_{b0}\times(\Delta V-I_{ss}\times R_{iss})) \qquad (2)$$

where I_0 and I_{ss} are initial and steady-state current, which were recorded by chronoamperometry for 4000 s. R_{bss} and R_{b0} are the initial and steady-state values of the bulk resistance. R_{i0} and R_{iss} are interfacial resistance between the electrode and electrolyte before and after the test. The Li||Li symmetric cells and LFP||Li full cells were charged and discharged on a LAND-CT3001A battery tester (Wuhan LAND Electronic Co. Ltd.). All electrochemical cell performances were tested under room temperature

Theoretical calculation

The geometric structures of all molecules are fully optimized by using the mixture B3LYP of 6-31G+ (d, p) basis set in Gauss software. The Density functional theory (DFT) was used to calculate the visualize the electrostatic potential maps, LUMO-HUMO energy level, and binding energy. In order to simplify the computational model, one repeating unit of one polymer chain was selected as the computational model for the polymer matrix. The effect of lithium anion on the system was ignored

in the calculation of the interaction between lithium ion and polymer. Very tight convergence criteria were adopted for all computational optimizations. In the binding energy calculation, the exchange correlation interaction is described by the generalized gradient approximation (GGA) and the Perdew-Burke-Ernzerhof (PBE) functional. For the construction of the surface model, a 15 Å vacuum was used to eliminate interactions between periodic structures. The adsorption energies (E_{abs}) of PAFP or PEO on the surface of LCO (001) were calculated as follows. $E_{abs} = E_{total}-E_{PAFP/PEO}-E_{LCO}$, where E_{total}, $E_{PAFP/PEO}$ and E_{LCO} are the energy of the whole system, the energy of PAFP or PEO molecules and the energy of the surface of LCO (001), respectively.

Supplement Figures

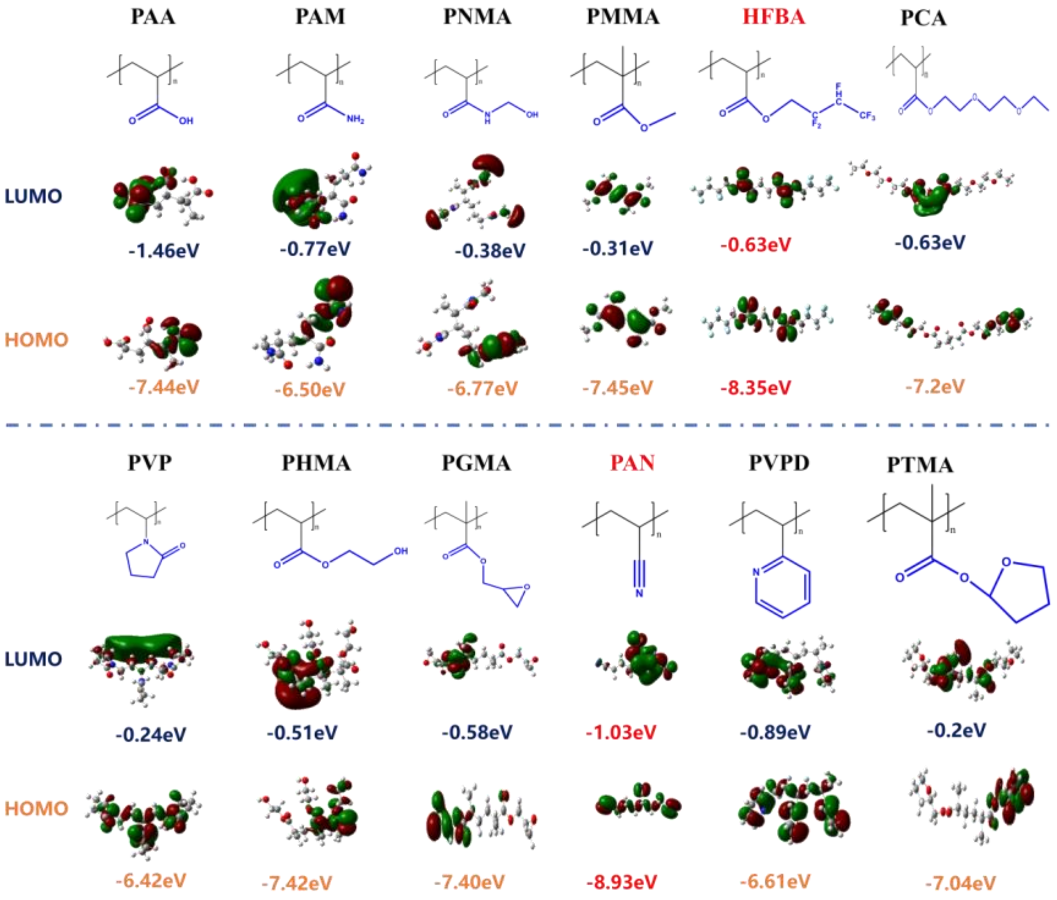

Figure S1. The HOMO and LUMO energy levels of different polymers.

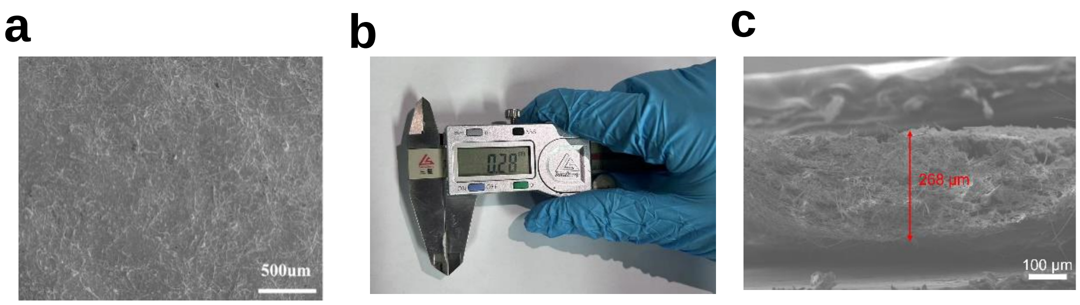

Figure S2. a) SEM plot of PAFP after in situ polymerization in the separator. b) Thickness of PAFP. c) Cross-sectional SEM images of PAFP.

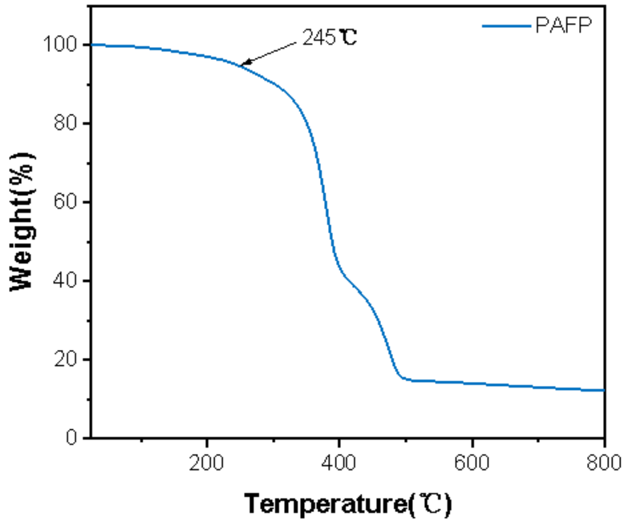

Figure S3. TGA curves of PAFP.

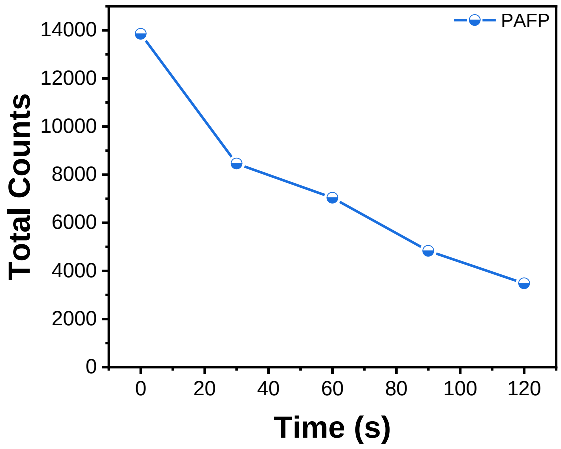

Figure S4. Total number of HO· radical radicals for PAFP.

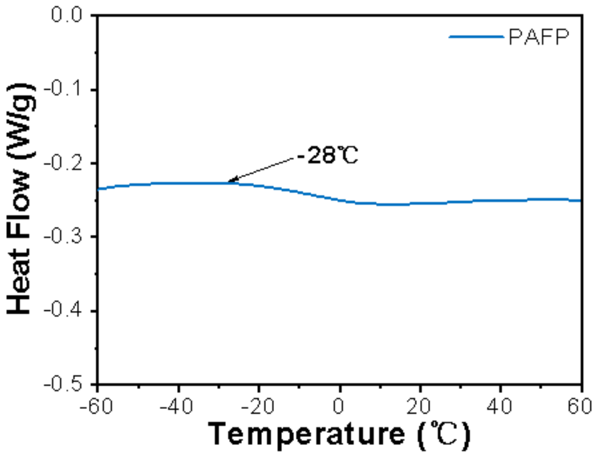

Figure S5. DSC curves of PAFP.

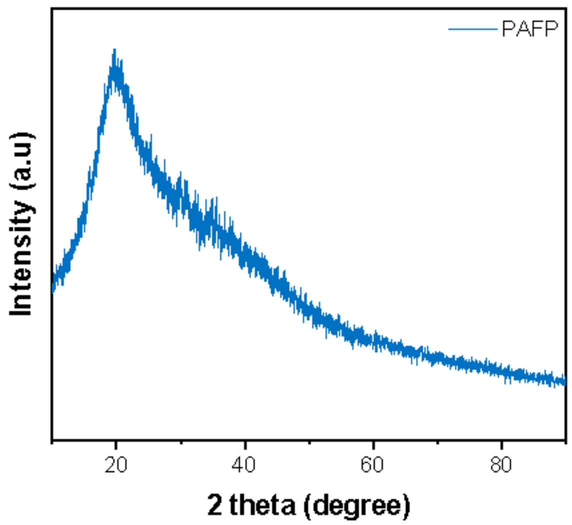

Figure S6. XRD curves of PAFP.

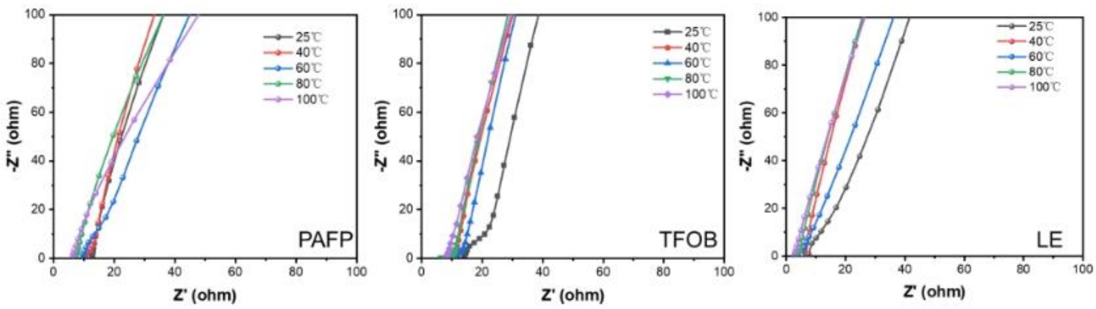

Figure S7. EIS plots of PAFP, TFOB and LE at different temperatures.

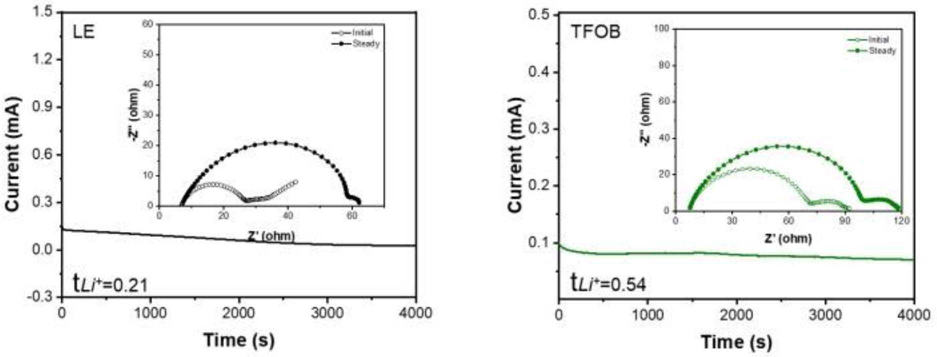

Figure S8. Polarization curves, initial and steady-state impedance maps of TFOB and LE at room temperature.

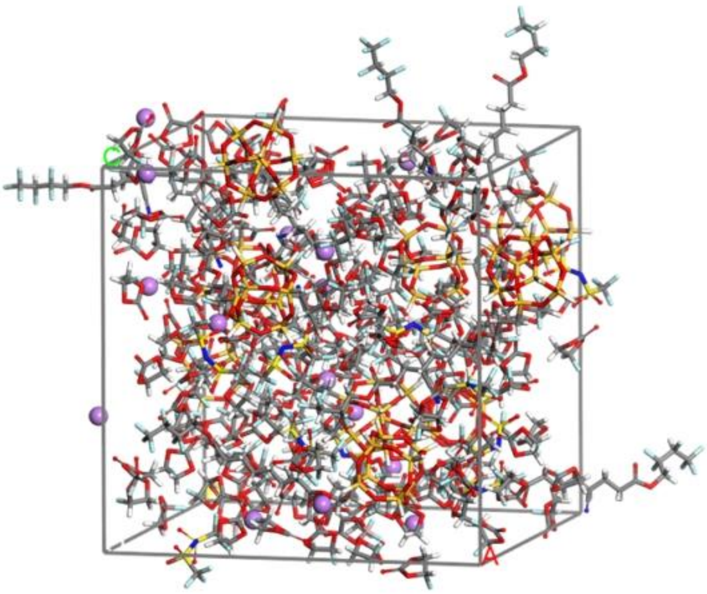

Figure S9. Snapshot of a Molecular dynamics (MD) simulation of PAFP.

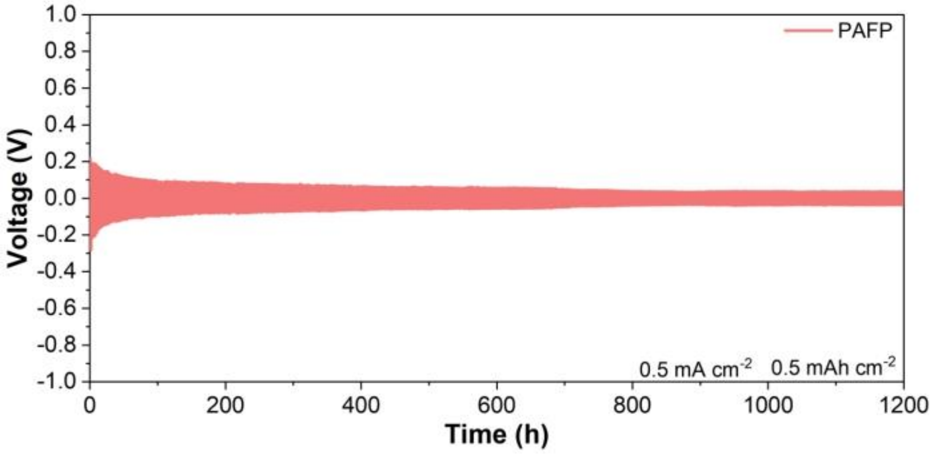

Figure S10. Galvanostatic cycling curves of Li||Li symmetric cells with PAFP at 0.5 mA cm^{-2}, 0.5 mAh cm^{-2}..

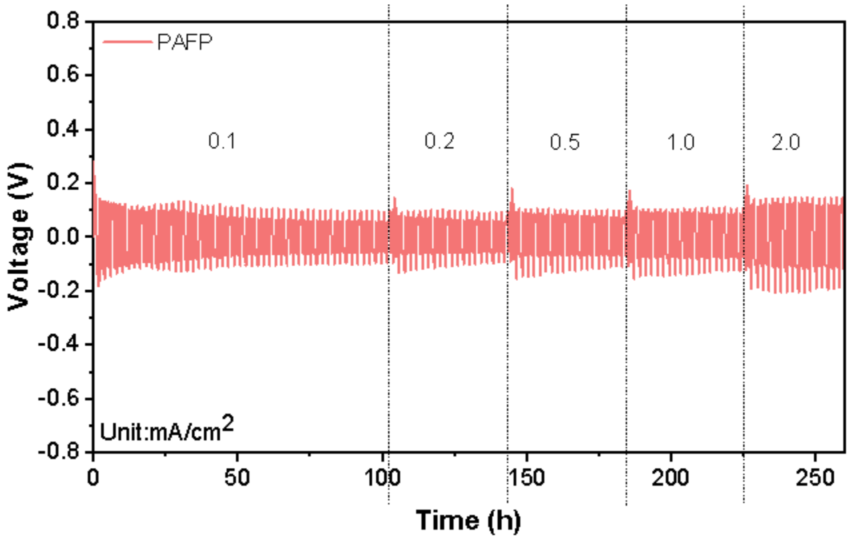

Figure S11. Constant current cycle curves of Li|PAFP|Li symmetric cells at different current densities.

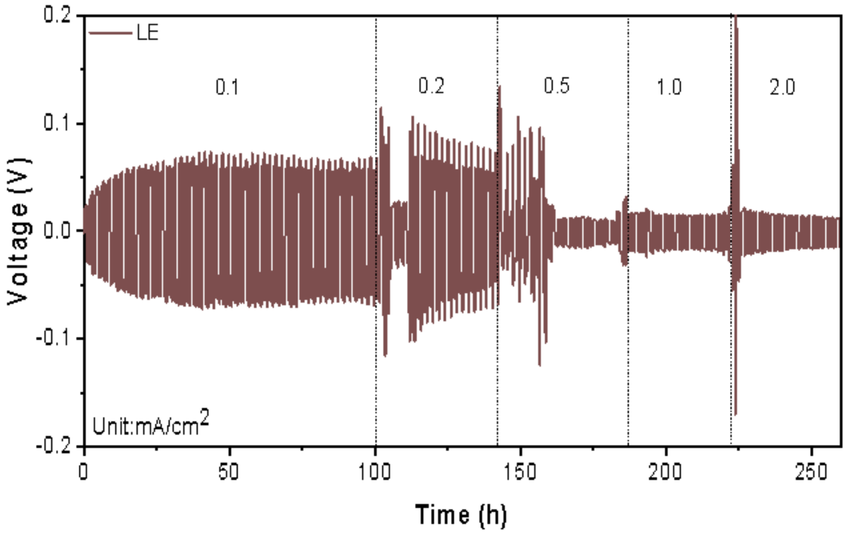

Figure S12. Constant current cycle curves of Li|LE|Li symmetric cells at different current densities.

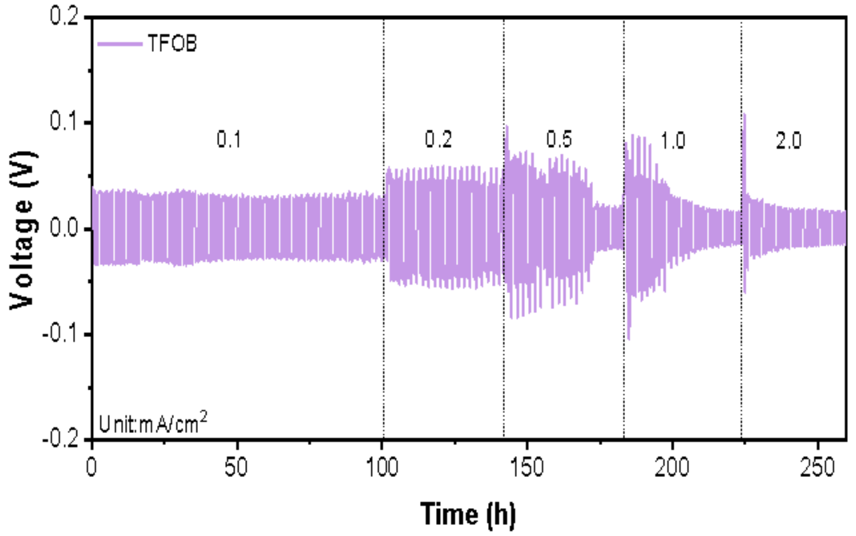

Figure S13. Constant current cycle curves of Li|TFOB|Li symmetric cells at different current densities.

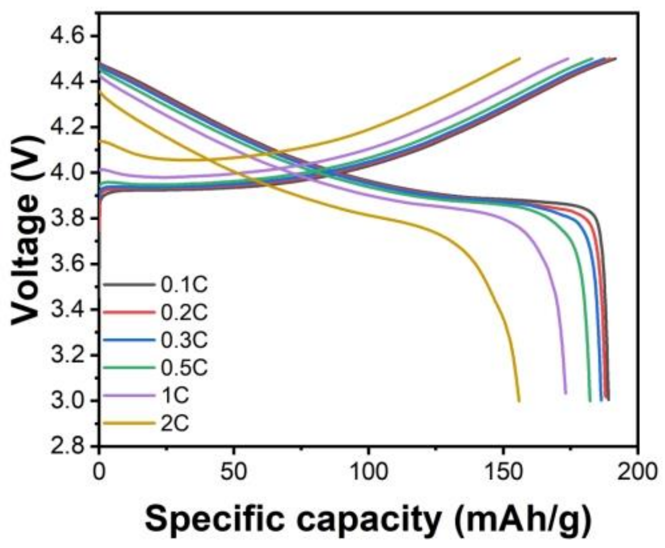

Figure S14. Charging and discharging curves of PAFP at 0.1-2 C multipliers.

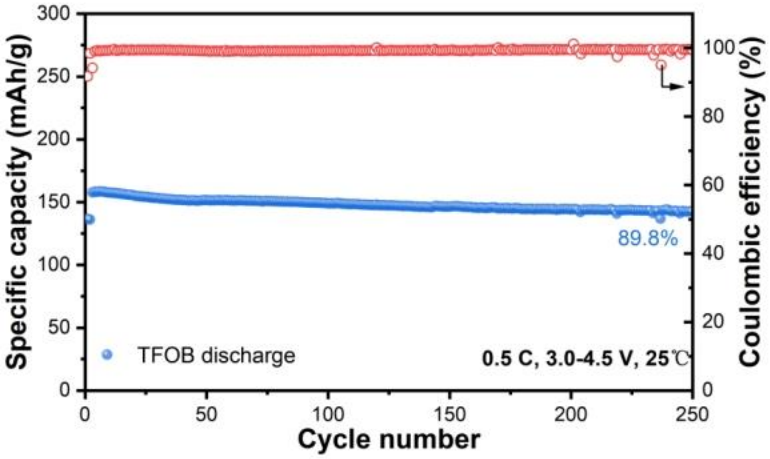

Figure S15. Long cycle performance of LCO|TFOB|Li cells at 0.5 C, 4.5 V.

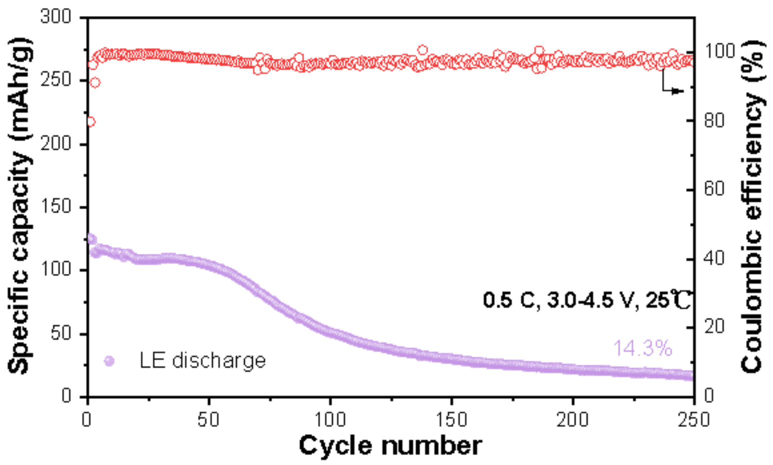

Figure S16. Long cycle performance of LCO|LE|Li cells at 0.5 C, 4.5 V.

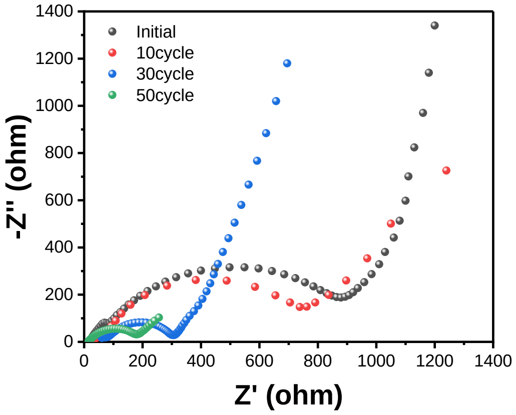

Figure S17. The EIS curve of Li|LCO cells with PAFP at before and after cycles.

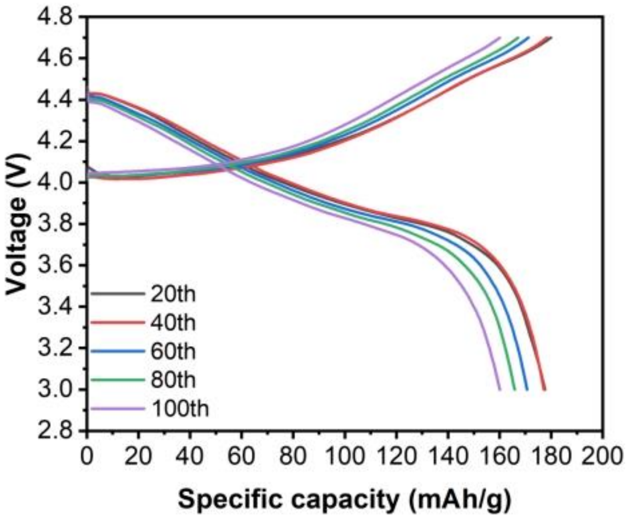

Figure S18. Charge and discharge curves of the LCO|PAFP|Li battery at 4.7 V.

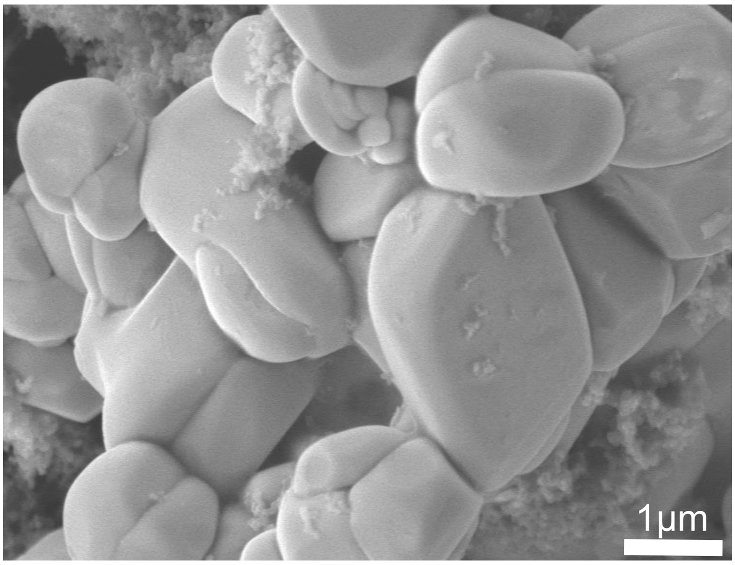

Figure S19. SEM images of LCO particles before cycling.

Table S1. Comparison of battery cycle performance between this paper and other papers.

Electrolyte	Rate (C)	Cycle number (cycles)	Battery system	Voltage range	Specific capacity (mAh g^{-1}) (Capacity retention)
**P-DOX**	1C	100	Li//LCO	2.8-4.5V	163.5 (92.9%)
**PVDF/PVAC-based CPE**	0.5C	200	Li//LCO	3.0-4.5V	190.8 (85%)
**CPCE**	0.5C	300	Li//LCO	3.0-4.3V	146 (94.2%)
**PCGPE10-1**	1C	270	Li//LCO	3.0-4.4V	138 (81%)
**SN-LiDFOB**	0.3C	200	Li//LCO	3.0-4.3V	140.5 (88%)
**PEO(16:1)/PEO(4:1)**	0.1C	100	Li//LCO	3.0-4.2V	108.6 (82.6%)
**PE-CPE**	0.2C	385	Li//LCO	3.0-4.2V	145.3 (76.1%)
**IPLL-SSE**	0.5C	120	Li//LCO	3.0-4.3V	128.8 (92.8%)
**Poly-DOL-40FEC-HDI**	0.5C	500	Li//LCO	3.0-4.2V	138 (80%)
**★This work**	**1C**<br>**1C**	**500**<br>**100**	**Li//LCO**<br>**Li//LCO**	**3.0-4.5V**<br>**3.0-4.7V**	**178.8 (90.4%)**<br>**174.3 (87.7%)**
